# Impact of timing of breastfeeding initiation on neonatal mortality in India

**DOI:** 10.1186/s13006-018-0162-0

**Published:** 2018-07-03

**Authors:** Deepika Phukan, Mukesh Ranjan, L. K. Dwivedi

**Affiliations:** 1 Department of Public Health and Mortality Studies, International Institute for Population Sciences, Mumbai, Maharashtra 400088 India; 2 Department of Mathematical Demography & Statistics, International Institute for Population Sciences, Mumbai, Maharashtra 400088 India

**Keywords:** Breastfeeding, Neonatal mortality, IDHS-II, Binary logistic regression, Population attributable risk

## Abstract

**Background:**

Neonatal mortality defined as a death during the first 28 days of life and is the most critical phase of child survival. In spite of the strong evidence supporting immediate and long term health benefits of timely initiation of breastfeeding in India, only two-fifths (44%) of children receive breastfeeding within 1 h of birth. This study aims to examine the role of a behavioral factor i.e., timing of initiation of breastfeeding on neonatal deaths.

**Methods:**

Data from India Human Development Survey-II (IHDS-II), 2011–12, a nationally representative, large scale population-based dataset has been used. Sample Registration System (SRS) has been used to examine the rate of change in Neonatal Mortality Rates from the year 2011 to 2015. District Level Household & Facility Survey (DLHS-4), 2012–2013 and Annual Health Survey(AHS), 2012–13 data have been used to show the district wise distribution of women who have breastfed their child within 1 h of birth. Population Attributable fraction has been computed using binary logistic regression model for various scenarios of breastfeeding within first hour of birth.

**Results:**

Less than one fourth (21%) of children were breastfed within 1 h of birth across the different districts of India, which varies from the lowest 15% in Sarasvati of Uttar Pradesh state to the highest 94.6% in Thiruvananthapuram of Kerala state. Findings suggest when women did not breastfeed their newborn within the 1 h after his birth, the odds of neonatal deaths were increased by nearly threefold (OR 2.93; 95% CI 1.89, 4.53) in comparison with those neonates who have breastfed within 1 h of birth. Population Attributable Risk estimates that the risk of the neonatal deaths could be reduced to a maximum of 15% when all babies would expose to early breastfeeding from the present level of breastfeeding.

**Conclusions:**

We found that timely initiation of breastfeeding is beneficial for child survival within the first 28 days of birth, including all causes of mortality. Therefore, efforts in formulating an effective policy focusing on early initiation of breastfeeding are needed.

## Background

Globally, around 5.6 million children died before reaching their fifth birthday, of those, 2.6 million (or 46%) died in the first 30 days of life [1]. Approximately 7000 newborns died every day, most of which occurred within first 7 days after birth, with about 1 million dying on the first day and close to 1 million dying within the next 6 days in 2016 [2]. Most of the neonates died in Southern Asia (39%), followed by sub-Saharan Africa (38%). Half of all newborn deaths occurred in the following five countries: India, Pakistan, Nigeria, the Democratic Republic of the Congo and Ethiopia [1]. Over the past 25 years, the age under-five mortality rate dropped from 93 deaths per 1000 live births in 1990 to 41 in 2016. In India, in the year 2015, infant mortality accounts for 37 infant deaths per 1000 live births, of which 67.8% infants (25 per 1000 live births) died in the first month of births [3]. In 2013, India recorded the highest absolute number of neonatal deaths of any country, nearly 0.75 million [4]. Despite a significant change in neonatal mortality over the years, progress has been inadequate towards achieving Millennium Development Goal 4 (MDG-4) [5]. In 2015, the Sustainable Development Goals (SDGs) have been introduced, seeking to achieve all the goals by 2030. Goal 3 of the Sustainable Development (SDG 3) is focused on promoting MDG-4 to reduce the under-five mortality by two thirds, between 1990 and 2015 and will continue beyond 2015, until neonatal mortality is at least as low as 12 per 1000 live births and under-5 mortality to at least as low as 25 per 1000 live births [6].

Various factors can effectively reduce neonatal mortality to greater levels, early initiation of breastfeeding is one of them and it has benefits for survival and beyond. The World Health Organization (WHO) has recommended that all neonates to be breastfed within 1 h of birth. The deleterious effects of infections related infant deaths can be prevented by early initiation of breastfeeding (or human milk feeding) and exclusive breastfeeding which is the easiest, cost-effective and life-saving intervention for the health of a newborn [7].

Early initiation of breastfeeding and exclusive breastfeeding for the first 6 months of life prevents around 20% newborn deaths and 13% under-five deaths [8]. It can also reduce mortality due to neonatal infections (sepsis, pneumonia, tetanus, and diarrhea) [9] which contribute 36% in neonatal deaths from all causes, and preterm birth an additional 27% [10]. In spite of the strong evidence supporting immediate and long term health benefits, early initiation of breastfeeding in South Asia remains low with varying rate with 36.4% in India, 24% in Bangladesh and 8.5% in Pakistan [11–13]. In India, only 65% of children are exclusively breastfed for the first 6 months and 45% of children receive breastfeeding within 1 h of birth, though breastfeeding is one of the most important interventions of child survival [14]. In the year of 2016, the Government of India launched the National Breastfeeding Promotion Programme MAA (mothers’ absolute affection) to ensure adequate awareness is generated among the masses, especially mothers, on the benefits of breastfeeding. The Programme will be implemented at three levels: Macro level through mass media; meso level in health facilities and micro level at communities [15].

Prior studies have shown that early initiation of breastfeeding is associated with a lower risk of neonatal mortality [16–19]. It is found that globally, only half of newborn babies are breastfed during their first hour of birth, despite strong evidence of nutritional and immunological benefits of early initiation in reducing neonatal mortality and morbidity [18, 20, 21]. The first milk (colostrum) contains bioactive immune factors which protect a neonate against a variety of infections and allergic diseases [22]. A recent systematic review of the literature based on 25 studies from seven countries in South Asia, revealed that early initiation of breastfeeding is predominately associated with socioeconomic, health related and individual factors, [23] and that insufficient attention is afforded to intrapartum and neonatal characteristics.

A few studies in India have examined associations between timing initiation of breastfeeding and neonatal mortality in different communities [24, 25]. There is a need for a national level study as it is important to examine the association between timing of initiation of breastfeeding and neonatal mortality nationally. The present study investigates the role of timing of initiation of breastfeeding in improving neonatal survival using a nationally representative large scale population-based dataset in India. In order to achieve the Sustainable Developmental Goals within stipulated time, we need to emphasize on the evidence-based conclusion regarding the linkages between timing of breastfeeding and neonatal mortality reduction as there is an urgent need to improve breastfeeding practices in India.

## Methods

The India Human Development Survey-II (IHDS-II), 2011–12, has been used for the present analysis, which is a nationally representative and multi-topic survey of 42,152 households in 1503 villages and 971 urban neighborhoods across India. Most of these households had been interviewed for IHDS-I (2005). Both surveys cover all states and union territories of India with the exception of Andaman/Nicobar and Lakshadweep. Sample Registration System (SRS) dataset is used to see the rate of change in NMR from the year 2011 to 2015. District Level Household & Facility Survey (DLHS-4), 2012–2013 and AHS (Annual Health Survey), 2012–13 state data have been used to show the district wise distribution of women who have breastfed their child within 1 h of birth.

### Definition of variables

#### Outcome variable

In the present analysis, neonatal mortality was taken as the outcome variable and coded as “0” for non-occurrence of neonatal death and “1” for occurrence of neonatal death. The analysis is restricted only for the last live births preceding the survey i.e. births since January 2005. A total of 37,350 births were recorded as last births in IHDS-II, 2011–12. Of a total live births, 340 children died before the completion of 28 days after birth.

#### Exposure variables

The independent variables for the study were divided into community, household, and maternal and child level variables. Initiation of breastfeeding is computed from the question that “how long after birth, the mother first put her child to the breast”. Early breastfeeding defined as a mother who put her child to the breast in less than an hour of his/her birth. The community level factors were state Regions and Place of residence (Rural/Urban). The data are available for all states and union territories of India with the exception of Andaman/Nicobar and Lakshadweep. The states were subdivided into five board regions as 1) North Region: Jammu & Kashmir, Himachal Pradesh, Punjab, Chandigarh, Uttarakhand, Haryana, Delhi, Uttar Pradesh, 2) West Region: Rajasthan, Gujarat, Daman & Diu, Dadar and Nagar Haveli, Maharashtra, 3) South Region: Andhra Pradesh, Karnataka, Goa, Kerala, Tamil Nadu, Puducherry, 4) Central Region: Chhattisgarh, Madhya Pradesh, 5) East Region: Bihar, Jharkhand, Odisha, West Bengal, Arunachal Pradesh, Manipur, Meghalaya, Mizoram, Nagaland, Tripura, Sikkim. As established in literature, the household level variables were Religion (Hindu/Muslim/Others), Caste (Scheduled Tribe/Scheduled Caste/Others), and Wealth Quintile. The wealth quintile was constructed from the total income of the household. It was categorized into five quintiles (Poorest/ Poorer/ Middle/Richer and Richest). The maternal level factors involved- Mother’s age, Mother’s education and Body Mass Index (BMI). The information was available in continuous which were converted into categorical variables with Mother’s age (15–24/25–34/35+), Mother’s education (No education/Primary/Secondary/Higher) and BMI (Underweight/ Normal/ Overweight /Obese). Child level variables like birth size (Large/Average/Small/ Very Small), Birth order (1/ 2–4/5+) and sex of the child (Boy/Girl) included in the model as dummies.

### Statistical analysis

Univariate, Bivariate and Multivariate analysis were used in the present study. At the univariate level, Maps were generated by using Arc-GIS (version 10.5) and Geo Da (1.12 version) software. The bivariate analysis examined the relationship between the selected community, household, maternal and child level factors and neonatal deaths. At the Multivariate Analysis, Binary Logistic Regression Model has been used. Population Attributable Risk (PAR) has been calculated post estimated after multivariate logistic regression model. STATA 14 has been used for the statistical analysis.

## Results

**Fig. 1:**
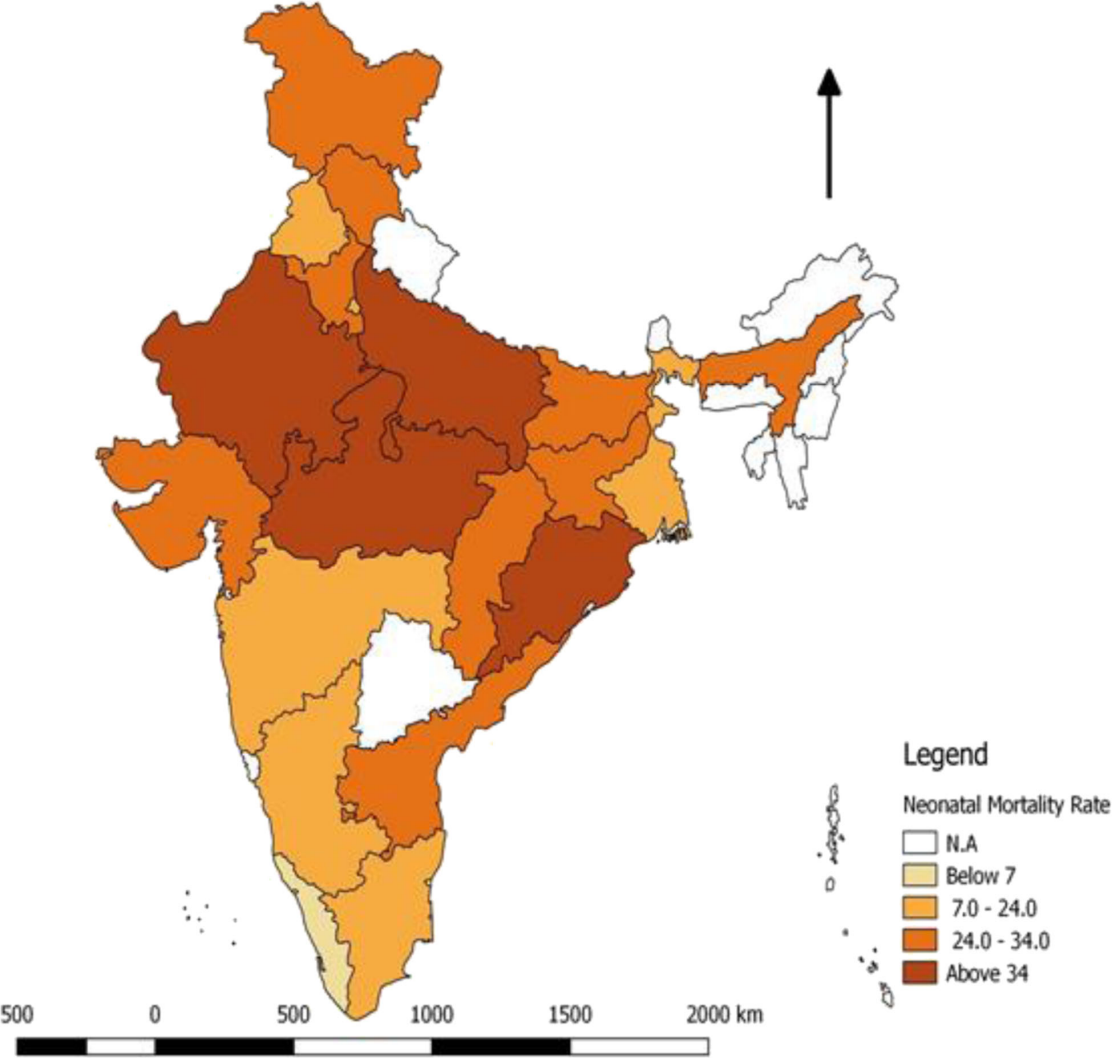
State wise Distribution of Neonatal Mortality Rate in India, 2011

**Fig. 2:**
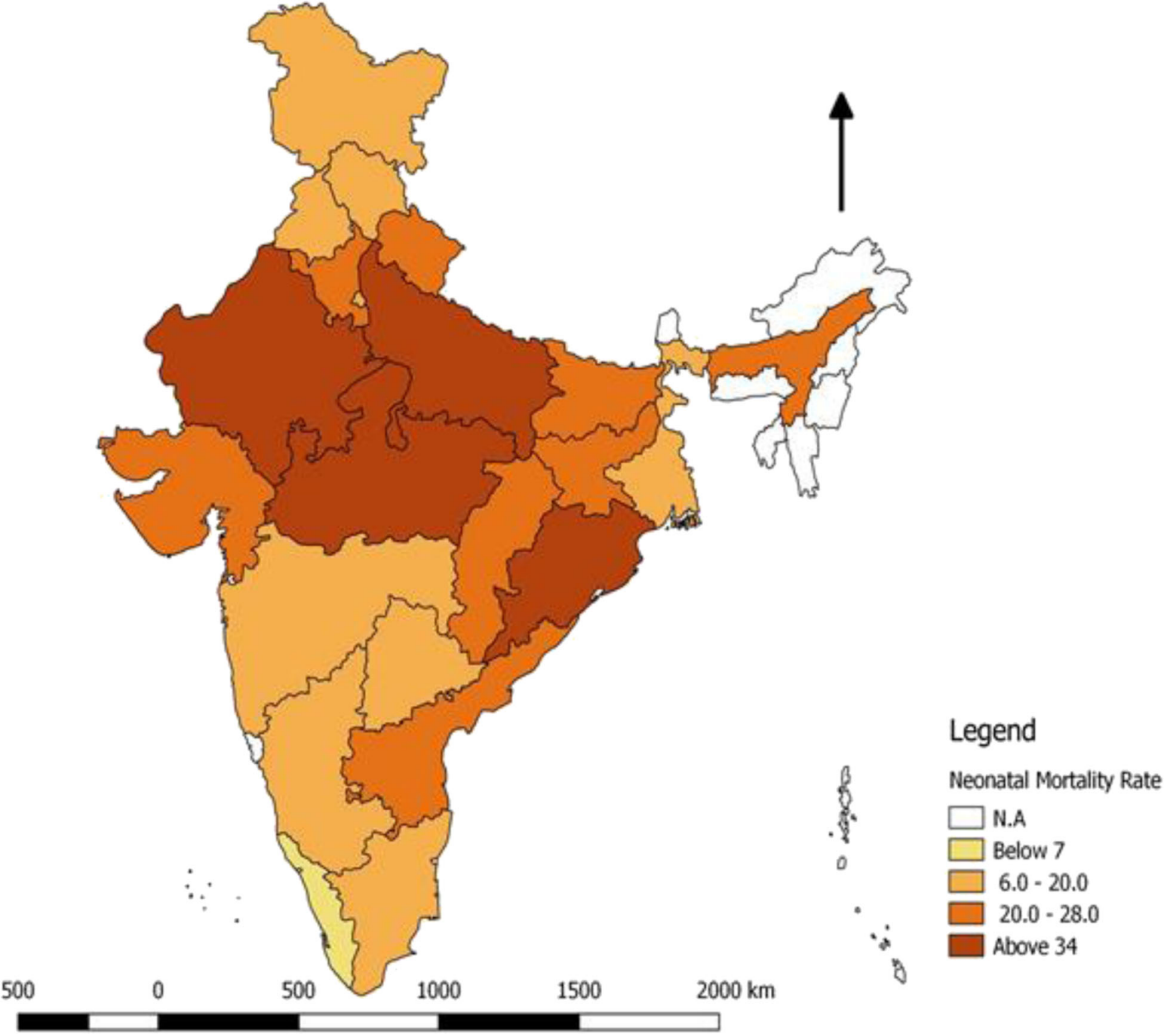
State wise Distribution of Neonatal Mortality Rate in India, 2015

Figures 1 and 2 show the State Wise Neonatal Mortality rates (NMR) in the year of 2011 and 2015. In 2011, six of the 22 states had an NMR of more than 30 per 1000 live births, which is the threshold level of Millennium Development Goal 4 for Neonatal Mortality Rate. By 2015, only three states had an NMR more than 30.

**Fig. 3:**
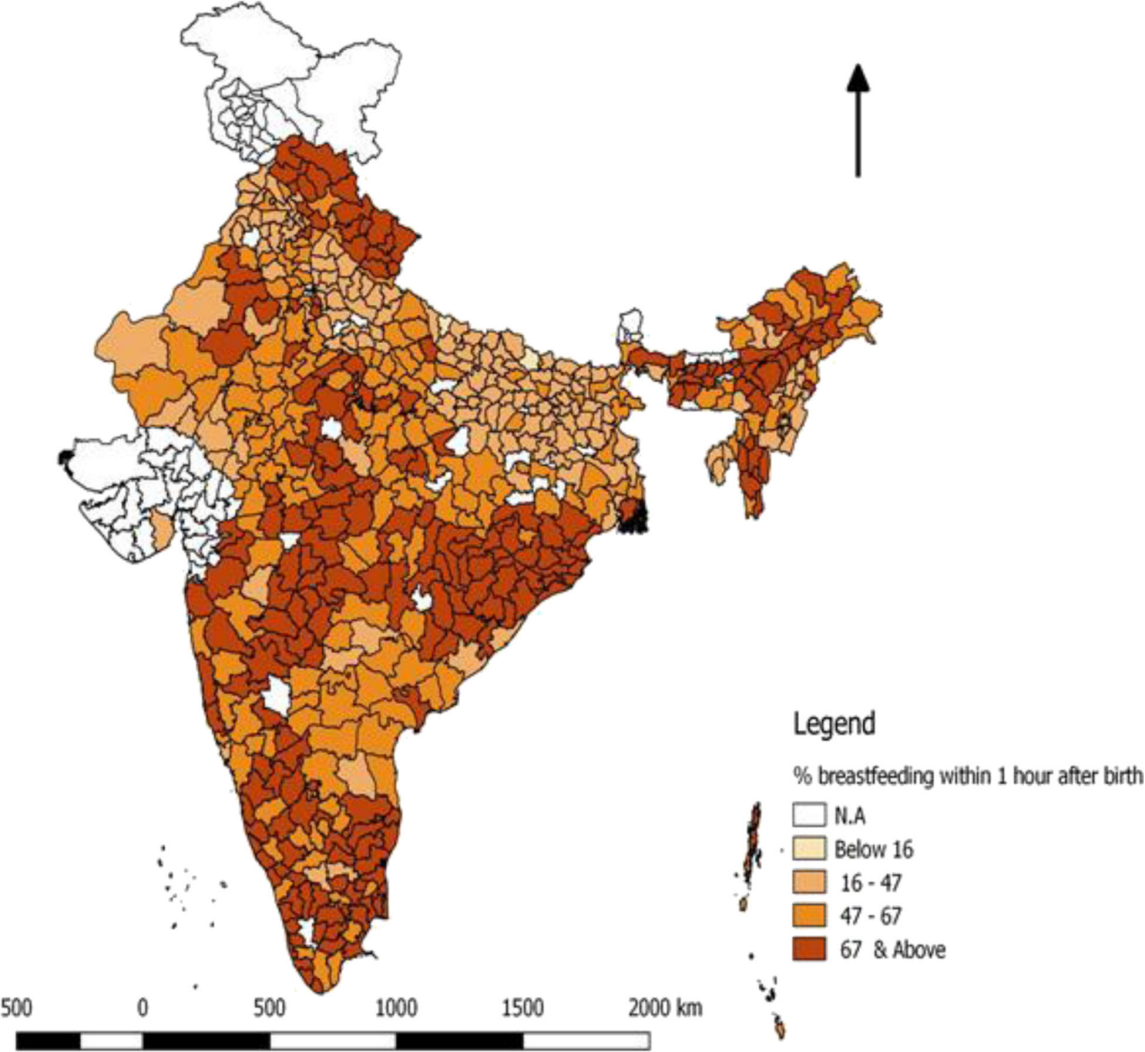
District wise prevalence of breastfeeding within 1 h of birth

From the Fig. 3, we have seen the district wise  prevalence of breastfeeding within 1 h of birth. The spatial variation showed less than 40% of women were practicing breastfeeding their babies within 1 h of birth, in some districts of Rajasthan, Punjab, Haryana, Bihar, Uttar Pradesh and North-East. One of the notable feature that emerges from the map is that many districts where the prevalence of practicing breastfeeding is low, lies mostly in Bihar and distributed uniformly across the state.

Table 1 gives the total number and percentage distribution of live births in accordance with the background characteristics. There were only 21% live births that have breastfed within the 1 h of birth. A majority of babies lived in rural areas, whereas more than three-fourths were Hindu dominant households and belong to Other Backward classes and Schedule tribes/caste. Maternal characteristics showed most of the babies were born to mothers in the age groups between 25 and 34 years. Only 11% of mothers had completed higher education. Less than half of the mothers were illiterate. Of the total number of mothers, only 3 % were obese and more than half belonged to normal BMI (56.83%). By examining the birth size of the babies, Table 1 depicts that almost 76% of babies were of average size at birth. The data shows an equivalent distribution of boys and girl across the nation.

**Table 1:** Distribution of neonatal deaths based on selected characteristics, India IHDS-II, 2011–12

Variables	Live births (%)	*N* = 37,350
Initiation of breastfeeding		
Early	21.1	7881
Delayed	78.9	29,477
Community level factors		
Interstate region		
North	32.62	12,185
West	18.58	6940
South	14.13	5277
Central	12.43	4644
East	22.25	8312
Place of residence		
Rural	73.34	27,399
Urban	26.66	9959
Household level factors		
Religion		
Hindu	76.56	28,602
Muslim	18.88	7055
Others	4.55	1701
Caste		
General	24.05	8986
OBC	41.55	15,523
ST/SC	34.39	12,849
Household Income		
Poorest	17.93	6664
Poorer	18.54	6890
Middle	20.34	7559
Richer	21.33	7928
Richest	21.86	8122
Maternal level factors		
Mother’s age		
15–24	13.87	5181
25–34	57.94	21,647
35+	28.19	10,530
Women’s education		
No Education	44.02	16,441
Primary	6.34	2366
Secondary	38.29	14,299
Higher	11.36	4242
BMI		
Underweight	28.36	10,353
Normal	56.83	20,745
Overweight	11.63	4247
Obese	3.17	1157
Infant level factors		
Birth size		
Large	8.16	2927
Average	75.53	27,093
Small	14.47	5189
Very Small	1.84	661
Birth order		
1st order	10.5	14,244
2–4 order	61.12	19,216
5+ order	28.38	3890
Sex of the child		
Boy	49.39	18,446
Girl	50.61	18,904

In Table 2, odds ratios were given with 95% of confidence interval after adjusting the covariates. If a woman did not breastfeed their newborn within 1 h after his/her birth then the odds of neonatal mortality is increased four (OR 3.54; 95% CI 2.34, 5.38) times compared to those neonates who have breastfed within 1 h of birth. Once controlling for all the community and household level variables in Model I, the risk was still significant. There was a significant difference in neonatal mortality between interstate regions. The odds of neonatal mortality are decreased by 40% (OR 0.60; 95% CI 0.42, 0.86) for the West region as compared to the Northern region. Variation can also be seen in the Northern and Southern region; neonates were 52% (OR 0.48; 95% CI 0.30, 0.75) less likely to die in the South than the North region. In the case of wealth quintile, neonates who belonged to the richest family has 48% (OR 0.52; 95% CI 0.35, 0.77) lower risk than those from the poorest family. When we add maternal and child level factors to Model II, Model III result showed that as the age of mother increases, neonatal deaths decreased. If a woman belongs to age 25–34, the odds of neonatal mortality is decreased by 46% (OR 0.54; 95% CI 0.39, 0.76) than those women who belong to age 15–24, further odds of neonatal mortality decreased to 49% (OR 0.51; 95% CI 0.33, 0.97) for the women belonging 35 years and above. Education levels of the mother also have a significant effect on neonatal mortality. Higher educated mothers have experienced less neonatal deaths than illiterate mothers. The expected odds of neonatal deaths is three times (OR 3.27; 95% CI 1.69, 6.34) higher for very small size babies than large size babies at birth. Neonatal death is decreased by 56% (OR 0.46; 95% CI 0.32, 0.67) for higher birth order children, and by 26% (OR 0.74; 95% CI 0.59, 0.94) for a girl child compared to a boy child.

**Table 2:** Risks of neonatal mortality according to time of initiation of breastfeeding, community level, household level, maternal and child level variables, IHDS-2, 2011–12

Variables	Model I, OR (95% CI)	Model II, OR (95% CI)	Model III, OR (95% CI)
Initiation of breastfeeding			
Early®	1	1	1
Delayed	3.54 (2.34, 5.38)**	3.49 (2.29, 5.33)**	2.93 (1.89, 4.53)**
Community level factors			
Interstate region			
North®		1	1
West	–	0.60 (0.42, 0.86)*	0.61 (0.41, 0.89)*
South	–	0.48 (0.30, 0.75)**	0.29 (0.16, 0.53)**
Central	–	0.92 (0.66, 1.29)	1.02 (0.72, 1.44)
East	–	1.22 (0.93, 1.60)	1.09 (0.80, 1.49)
Place of residence			
Rural®	–	1	1
Urban	–	0.77 (0.58, 1.03)	0.84 (0.61, 1.15)
Household level factors			
Religion			
Hindu®	–	1	1
Muslims	–	1.128 (0.83, 1.53)	0.88 (0.61, 1.26)
Others	–	0.93 (0.54, 1.62)	1.21 (0.69, 2.13)
Caste			
General®	–	1	1
OBC	–	1.13 (0.83, 1.52)	1.36 (0.95, 1.92)
ST/SC	–	1.31 (0.94, 1.81)	1.45 (1.00, 2.12)
Wealth quintile			
Poorest	–	1	1
Poorer	–	0.71 (0.52, 0.99)	0.86 (0.60, 1.23)
Middle	–	0.87 (0.64, 1.19)	1.02 (0.72, 1.45)
Richer	–	0.76 (0.55, 1.07)	0.89 (0.62, 1.29)
Richest	–	0.52 (0.35, 0.77)**	0.67 (0.44, 1.04)
Maternal level factors			
Mother’s age			
15–24®	–	–	1
25–34	–	–	0.54 (0.39, 0.76)**
35+	–	–	0.51 (0.33, 0.97)*
Women’s education			
No education®	–	–	1
Primary	–	–	0.46 (0.23, 0.91)*
Secondary	–	–	0.97 (0.73, 1.29)
Higher	–	–	0.55 (0.31, 0.99)*
BMI			
Underweight®	–	–	1
Normal	–	–	0.89 (0.68, 1.16)
Overweight	–	–	1.37 (0.91, 2.06)
Obese	–	–	1.73 (0.90, 3.30)
Infant level factors			
Birth size			
Large®	–	–	1
Average	–	–	0.92 (0.57, 1.47)
Small	–	–	1.45 (0.86, 2.43)
Very Small	–	–	3.27(1.69, 6.34)**
Birth order			
1st order®	–	–	1
2–4 order	–	–	0.46 (0.32, 0.67)**
5+ order	–	–	0.71 (0.45, 1.14)
Sex of the child			
Boy®	–	–	1
Girl	–	–	0.74 (0.59, 0.94)*

® Reference Category, **p < 0.01, *p < 0.05

Table 3 provide the post estimated value of logit model for Population Attributable Fraction (PAF) for different situations of breastfeeding. In the Table 3, the Population Attributable Risk was estimated for the situation when all babies are exposed to breastfeeding. Scenario 0 represents the baseline scenario which is an overall predicted probability of neonatal deaths at adjusted level while, Scenario 1 (fantasy scenario) represents the predicted probability of neonatal deaths when all babies are exposed to breastfeeding and other factors in the model are adjusted. In this study, we found PAF to be − 0.15 which indicates that when all babies are exposed to early breastfeeding, the risk of the neonatal deaths could be reduced to a maximum of 15% from the present level. Similarly, in Table 4 we have chosen a scenario (Scenario 1) when all the babies were not breastfed within the first hour of birth We found that in this extreme worst situation, the risk of neonatal deaths will increase to the level of 60%.

**Table 3:** Population Attributable Risk when all babies are exposed to breastfeeding

	Mean/Ratio	[95% CI]	
Scenario_0	0.008**	0.007	0.009
Scenario_1	0.009**	0.008	0.011
PUF	1.151**	1.108	1.196
PAF	−0.151**	−0.196	−0.108

**p < 0.01

**Table 4:** Population Attributable Risk when all babies are not exposed to breastfeeding

	Mean/Ratio	[95% CI]	
Scenario_0	0.008**	0.007	0.009
Scenario_1	0.003**	0.002	0.009
PUF	0.398**	0.268	0.593
PAF	0.601**	0.407	0.732

**p < 0.01

## Discussion

The present study supports the protective effect of early breastfeeding initiation on death within the first 28 days, including all-cause mortality. Wide interstate and intrastate variations exist in neonatal mortality across the country [26]. The present study also depicts that there is a north-south variation seen in neonatal deaths. It is clear from the maps that northern part of India is more vulnerable to neonatal mortality than southern part of India. Neonatal deaths can be prevented or reduced by early initiation of breastfeeding and exclusive breastfeeding [7]. There is a stark variation seen in children who have breastfed within 1 h of birth across the districts. The districts of Kerala, Tamil Nadu, Maharashtra, Odisha, Chhattisgarh, Madhya Pradesh, Himachal Pradesh, Uttarakhand and some districts of the North-East babies are more exposed to early initiation of breastfeeding, while babies from the districts of Bihar, Uttar Pradesh, Haryana, and Punjab are more exposed to delayed initiation of breastfeeding, where the rate of neonatal deaths are also high.

Newborn care immediately after the birth is vital since 40% of all neonatal deaths occur on the first day of life and 56% during the first 3 days [27]. The timing of breastfeeding initiation is found to be the most significant risk factor which affects neonatal deaths [7]. Early initiation of exclusive breastfeeding serves as the starting point for a continuum of care for mother and newborn that can have long-lasting effects on health and development [28]. The findings of the present study resemble its predecessor. There was an odd of 3-fold increase in the risk of neonatal deaths if there was a delay in initiation of breastfeeding.

In India, there are wide disparities in mortality by caste, region, place of residence, and economic status, among other characteristics [29]. These variations are related to differences in wealth, nutrition, education, availability of health services, culture and gender equality status [30]. The literature suggests that infant mortality rates have been inversely related to socioeconomic status, and child mortality is higher among the low-income families than non-poor families [31], which is consistent with our study.

Maternal and child level determinants as the weight of neonate, gestational age, and age of mother play a major role in the neonate’s survival [32]. Also, consistent with the earlier findings, maternal factors like mother’s age and education were prominent factors in neonatal mortality. Along with the maternal characteristics, child level factors have a notable effect on neonatal mortality. Very small babies were more likely to die than larger babies, babies and males were more vulnerable to neonatal death than females.

It is also found that two-thirds of the incidence of neonatal mortality can be attributed to delayed breastfeeding i.e., out of the total population, 60% of the babies died within 1 month of their birth because of they were not breastfed within 1 h. It has strong policy implications as we could reduce the risk of neonatal deaths to a great extent by giving them timely breastfeeding.

The limitations of the present study are that IHDS-II data were self-reported, as information on each outcome and determinants were collected retrospectively, there could be recall bias, that may have impact on results.

## Conclusions

Though India has witnessed momentous changes in the infant health scenario over the years, the changes have not been uniform. Neonatal deaths are influenced by host of variables like community, household, maternal and infant level factors. The early initiation of breastfeeding can reduce the risk of neonatal deaths by odds of three times. So, these findings support the recommendations of early initiation of breastfeeding as an intervention to reduce neonatal mortality. The implementation of policies and pro-breastfeeding routines are the major recommended interventions to achieve SDGs to reduce neonatal mortality.

## Abbreviations

AHS: Annual Health Survey
BMI: Body Mass Index
CI: Confidence interval
DLHS: District Level Household & Facility Survey
IHDS: The India Human Development Survey
IMR: Infant mortality rate
MDGs: Millennium Development Goals
NMR: Neonatal Mortality Rate
PAR: Population Attributable Risk
SDGs: Sustainable Development Goals
SRS: Sample Registration System
WHO: World Health Organization
